# Characteristics of facilities with specialized programming for drinking drivers and for other criminal justice involved clients: analysis of a national database

**DOI:** 10.1186/1747-597X-2-26

**Published:** 2007-08-30

**Authors:** Cynthia L Arfken, Sheryl Pimlott Kubiak

**Affiliations:** 1 Department of Psychiatry and Behavioral Neurosciences, Wayne State University, 2761 E. Jefferson, Detroit, Michigan, USA; 2 School of Social Work, Michigan State University, 254 Baker Hall, East Lansing, Michigan, USA

## Abstract

**Background:**

Offering specialized programming at substance abuse treatment facilities can help diversify clientele and funding sources, potentially enhancing the facilities' ability to survive and/or expand. Past research has shown that facilities only offering specialized programming for driving under the influence/driving while intoxicated offenders (DUI) are predominately private-for-profit owned. As criminal justice populations, both DUI and other criminal justice offenders, comprise a large proportion of those in community-based substance abuse treatment knowing facilities' characteristics would be important for administrators and policymakers to consider when updating programming, training staff or expanding capacity to ensure efficient use of scarce resources. However, while such characteristics are known for DUI programs, they are not known for facilities offering specialized programming for other criminal justice offenders.

**Methods:**

Analysis of the 2004 US National Survey of Substance Abuse Treatment Facilities.

**Results:**

Almost half the facilities (48.2%) offered either DUI or other criminal justice specialized programming. These facilities were divided between those offering DUI specialized programming (17.7%), other criminal justice specialized programming (16.6%) and both types of programming (13.9%). Certain characteristics were independently associated with offering DUI specialized programming (private ownership, rural location, for profit status) or other criminal justice specialized programming (receiving public funds, urban location, region of country).

**Conclusion:**

Offering specialized programming for DUI or other criminal justice offenders was common and associated with distinct characteristics. These observed associations may reflect the positioning of the facility to increase visibility, or diversify clientele and possibly funding streams or the decision of policymakers. As the criminal justice populations show no sign of decreasing and resources are scarce, the efficient use of resources demands policymakers recognize the prevalence of these specialized programming, join forces to examine them for efficacy, and explicitly incorporate these characteristics into strategies for workforce training and plans for treatment expansion.

## Background

Offering special programs for a target client population may reflect administrative decisions, explicit or implicit, to focus on a market, increase visibility, or diversify clientele and possibly funding streams. Alternatively, offering specialized programming may reflect requirements of policymakers. One possible target market is clients involved with the criminal justice system, as highlighted in a series of recent articles [1]. As has been clearly documented, substance abuse treatment for these clients both in institutional settings and in community-based facilities is an important component of efforts to reduce recidivism and the burden of substance abuse to society [2-5]. In addition, criminal justice referrals represent a substantial source of clients and revenue for community-based treatment facilities [6]: in fact, over a third of publicly funded admitted clients were referred by criminal justice entities, and the number of clients is growing at a faster rate than the overall treatment population [7].

Within the criminal justice population, there are distinct markets. One such market is people convicted of a DUI/DWI (Driving Under the Influence or Driving While Intoxicated, hereafter referred to as DUI) offence. This group has historically been viewed as distinct from other criminal justice offenders both for programming at substance abuse treatment facilities and by the federal government as a separate referral source to treatment. DUI offenders have been estimated to be 10% of criminal justice referrals [7]. As there were over 1.3 million DUI arrests in 2005 [8] covering misdemeanor and felony charges, this group would create a considerable demand for assessment and then in descending order education and formal treatment irrespective of conviction rate. This demand for service across the continuum can translate into significant revenue for facilities.

However, the distinction among criminal justice populations may also indicate other differences. For example, a recent report [9] focused on DUI programs in substance abuse treatment facilities found that facilities operating only DUI programs were owned predominately by private-for-profit enterprises (69%). This level of ownership was considerably higher than that for facilities operating both DUI programs and other types of programs (37%). The report did not speculate on why facilities with only DUI programs had such a high prevalence of private-for-profit ownership nor did it examine facilities with programming for other criminal justice populations to see if there was a similar ownership pattern.

One speculation for the attraction of DUI to private-for-profit owners would be payment differences based on revenue sources. Treatment services for other criminal justice populations receiving community-based services may be reimbursed through publicly funded systems, contracts from criminal justice entities such as the Department of Corrections or drug courts [10] which may have lower reimbursement rates than self pay or third party insurers. For the DUI offenders, third-party payers may refuse to fund services (of any kind), leading to self-pay [11]. There is, however, considerable economic diversity within the DUI population and some people rely on the publicly funded system for formal treatment [5,12].

There also may be increased societal or political pressure to provide specialized programming for other criminal justice populations in publicly owned facilities; alternatively, for-profit facilities may be attracted to increasing and diversifying their source of clientele. Location (i.e., region and population density) may be associated with offering specialized programming as the organization and financing of substance abuse treatment, especially its interface with the criminal justice system, varies by location. Rural locations, moreover, may incur lower costs facilitating expansion for additional programming or less pressure to specialize. Policymakers, however, may want to concentrate public funds paying for community-based treatment of other criminal justice offenders in urban areas where a larger proportion of offenders return to their home communities. All of these pressures may result in distinctive distributions between specialized programming for DUI offenders and other criminal justice offenders.

Opposing these distinctive distributions, facilities offering DUI programming have experience interfacing with criminal justice institutions such as courts so they may be more likely to offer specialized programming to other criminal justice populations. Specialized programming within substance abuse treatment in general considers the unique needs of a particular population and uses what is shown, or believed, efficacious in treating that need. Ideally specialized programming for clients referred by the criminal justice system would vary based on the needs associated with where they are along the criminal justice continuum, such as probation versus parole [13] and presence of other problems. Unfortunately given the considerable variation in treatment requirements across states [11], it is not possible to generalize what treatment people referred for DUI receive in treatment facilities. This variability also precludes knowing how DUI treatment may differ from other clients involved in the criminal justice system.

Previous investigators have examined characteristics of community-based substance abuse treatment facilities associated with specialized programming using annual surveys of U. S. substance abuse treatment facilities [e.g., [14-17]] including a report by Montoya [18] who examined for-profit status and offering core and auxiliary services. These surveys have the advantage of uniform data collection across a national census of facilities.

Building upon these studies, this paper examines characteristics of treatment facilities offering specialized programming targeted to the other criminal justice populations in general or those that target DUI services specifically. To do this, we examined the characteristics of facilities using a survey of all treatment facilities in the United States who either receive public funds or who wish to have their facilities publicized on the Substance Abuse Locator. The Substance Abuse Locator is an online listing of available substance abuse treatment facilities searchable by location and services offered. Within the locator, two specific searchable services are 1) specialized programming for DUI, and 2) criminal justice (other than DUI) clients. Although the distinction between DUI and other criminal justice involved clients may not always be clear, the currently configured online service offers the possibility of separate marketing strategies which may be associated with different facility characteristics.

## Methods

The public access data source was the 2004 National Survey of Substance Abuse Treatment Facilities (N-SSATS), a survey of treatment facilities from a continuously updated list of substance abuse treatment facilities [19]. The 2004 survey was used to be consistent with the report by DASIS on facilities with DUI/DWI programs [9]. When the survey was initially introduced it emphasized publicly funded programs and included sections on staff and client demographics. Since 2000, it has shed the staff and client demographic section and has vigorously expanded to all facilities offering substance abuse treatment. The inclusiveness of the list has been validated in one mid-size city [20]. The list includes facilities licensed, certified or monitored by the state substance abuse agencies for substance abuse treatment, but importantly, also private for-profit, small group practices, and hospital-based programs. For the year we examined they had a 96% participation rate. The updated list of facilities is used to provide immediate online assistance in locating substance abuse treatment facilities for the general public. As such, facilities without state funding also have a motivation to be included in the survey. To prepare for this survey, every year the files from the American Business Index and the American Hospital Association are reviewed for additional facilities. The state substance abuse agency approves the final list. Treatment facilities identified by the Bureau of Prison are not included.

The surveys were sent out 16,651 publicly and privately funded facilities with data collection spanning March 31 to October 1, 2004. Of the eligible facilities, 96% returned surveys. According to the report, facilities were eliminated due to closure, not providing treatment or services on March 31, 2004, other facility had reported their data already, providing treatment to incarcerated prisoners only or consisting of only a solo practitioner (n = 2,585).

Each facility was asked to report on the acceptance of different populations and the provision of different specialized programming from a pre-determined list. None of the programs were defined nor were there inquiries on how many clients were enrolled in any of them. The instructions did dictate, however, that programming refers to **specially designed **(underlined in the original) or group **exclusively for that type of client **(underlined in the original). For other criminal justice specialized programming, the item read "criminal justice clients (other than DUI/DWI clients)" [DWI = Driving while intoxicated.]. For DUI specialized programming, the item read "does this facility offer a special program for DUI/DWI or other drunk driver offenders at this location?" A separate question, immediately following, asked, "Are ALL of the substance abuse treatment clients at this facility enrolled in the DUI/DWI program?"

Other measures used in this analysis included ownership status, county of location, region, modality and revenue sources. For profit status, the question asked, "Is this facility operated by ... [a private for-profit organization]?" For public ownership, the question asked, "Is this facility operated by ... [State government, Local, county or community government, Tribal government, Federal Government]?" County of location was coded for the individual metropolitan statistical area (MSA) and a 9999 code for non-MSA. This latter code was used to indicate outside of a MSA (or non-MSA). Region had been coded as four categories (Northeast, Midwest, South and West) using U.S. census convention. Non-hospital residential treatment modality was coded yes if the facility answered yes to "Does this facility offer any of the following RESIDENTIAL (non-hospital) substance abuse services at this location [Residential detoxification, Residential short-term treatment (30 days or less), Residential long-term treatment (more than 30 days)]". Outpatient treatment was coded yes if the facility to "Does this facility offer any of the following OUTPATIENT substance abuse services at this location" [Outpatient detoxification, Outpatient methadone maintenance, Outpatient day treatment or partial hospitalization program (20 or more hours per week), Intensive outpatient treatment (defined as a minimum of 2 hours per day on 3 or more days per week), Regular outpatient treatment (fewer hours per week than intensive)]. For public funding, the question was: "Does this facility receive any public funds such as federal, state, county, or local government funds for substance abuse treatment programs? Do not include Medicare, Medicaid, or federal military insurance."

Of the 13,454 facilities in the public file, facilities offering DUI programming only (n = 260) and those with missing values for other criminal justice and DUI specialized programming or located outside the 50 states (n = 1656) were excluded. After these exclusions, there remained 11,538 facilities for analysis (85.8%).

To examine the associations between offering specialized programming and facilities' characteristics, multinomial logistic regression models in StataSE9.2 (Stata Corp., College Station, TX) were used with facilities not offering DUI or other criminal justice specialized programming as the reference category. Because regulation and funding decisions are at the state-level, potential impact of clustering within state was controlled by designating state as the "primary sampling unit". However, controlling for clustering reduces the power of the analysis so we repeated the analysis ignoring clustering as in Schultz et al. [14] and Mojtabai,[15]. In this latter analysis, we found region to be a significant predicator in the models. Associations are presented as odds ratios (OR) with 95% confidence intervals (95% CI). To test the stability of our responses, we repeated the analysis in the 2003 survey with virtually similar results (not shown). As there are no identifiers and the database is public access, the analysis was not considered human research by Wayne State University and therefore not reviewed by the institutional review board.

## Results

All facilities in the sample reported they accept criminal justice clients. Almost half the facilities (48.2%) offered either DUI or other criminal justice specialized programming. These latter facilities were divided between those offering DUI specialized programming (17.7%), other criminal justice specialized programming (16.6%) and both DUI and other criminal justice specialized programming (13.9%).

The facilities showed marked differences in prevalence by specialized programming offered (Table 1). For the bivariate associations (controlling for clustering within state) all the associations (tested using design based F-statistic) were highly significant (P < 0.0001) except ownership (public versus private) and region. For those two variables, the results were non-significant.

**Table 1:** Distribution of selected facility characteristics by specialized programming offered

	Type of specialized programming, %			
	DUI	Other CJ	DUI and other CJ	Neither

	(n = 2,063)	(n = 1,898)	(n = 1,619)	(n = 5,958)

For-profit status				
Yes	34.5	18.1	38.7	19.7
No	65.5	81.9	61.3	80.3
Public funds				
Yes	63.2	78.7	67.2	68.7
No	36.8	21.3	32.8	31.3
Public ownership				
Yes	8.9	11.0	12.2	11.9
No	91.1	89.0	87.8	88.1
Region				
Northeast	17.0	21.0	17.7	23.6
Midwest	31.3	19.4	23.5	24.1
South	29.2	23.2	29.1	26.6
West	22.5	36.4	29.7	25.7
Non-Metropolitan Statistical Area locations				
Yes	38.6	15.1	32.7	20.1
No	61.4	84.9	67.3	79.9
Outpatient treatment				
Yes	96.2	71.9	97.3	59.0
No	3.8	28.1	2.7	41.0
Non-hospital residential treatment				
Yes	9.3	35.4	8.9	36.3
No	90.7	64.6	91.1	63.7

DUI = Driving under the influence

CJ = other criminal justice

All comparisons were significantly different after controlling for clustering within states at p < 0.001 except ownership and region.

When examining the number of clients in treatment on the count day, there were marginal differences across the groups for hospital based treatment (F = 2.39, df = 3, 383, p = 0.07) and statistically significant (F = 13.27, df = 3, 7855, p < 0.001) but clinically small differences in mean number (and large variability) of clients from outpatient facilities offering DUI specialized programming (M = 88.7, SD = 127.7) to outpatient facilities offering both DUI and other criminal justice specialized programming (M = 118.8, SD = 144.9).

After adjustment for all other variables in the model (Table 2), receiving public funds increased the likelihood that a facility would offer other criminal justice specialized programming either with DUI or without DUI specialized programming. Private ownership increased the likelihood that a facility would offer DUI specialized programming; public ownership did not increase the likelihood of offering either of these specialized programming. For modality, outpatient treatment increased the likelihood of offering DUI or criminal justice specialized programming.

**Table 2:** Adjusted odds ratios and 95% confidence intervals between facilities' characteristics and type of specialized programming offered

	Type of specialized programming,									
	
	DUI			Other CJ			DUI and other CJ			Neither
	
	(n = 2,063)			(n = 1,898)			(n = 1,619)			(n = 5,958)
	
	OR	CI	CI	OR	CI	CI	OR	CI	CI	
For – profit status										
Yes	2.16	1.64	2.85	1.13	0.92	1.38	3.07	2.37	3.96	Reference
No	1.0			1.0			1.0			
Public funds										
Yes	1.11	0.90	1.38	1.84	1.41	2.42	1.62	1.29	2.02	Reference
No	1.0			1.0			1.0			
Public ownership										
Yes	0.63	0.44	0.90	0.85	0.70	1.03	0.97	0.65	1.45	Reference
No	1.0			1.0			1.0			
Region										
Northeast	0.97	0.33	2.83	0.62	0.47	0.81	0.74	0.28	1.92	Reference
Midwest	1.15	0.40	3.29	0.52	0.37	0.72	0.63	0.24	1.67	Reference
South	1.27	0.44	3.69	0.63	0.48	0.83	0.92	0.33	2.55	Reference
West	1.0			1.0			1.0			
Non-Metropolitan Statistical Area locations										
Yes	2.37	1.71	3.28	0.68	0.55	0.85	1.78	1.28	2.46	Reference
No	1.0			1.0			1.0			
Outpatient treatment										
Yes	12.24	8.63	17.37	2.52	2.19	2.89	19.84	14.35	27.43	Reference
No	1.0			1.0			1.0			
Non-hospital residential treatment										
Yes	0.56	0.43	0.73	1.39	1.01	1.91	0.58	0.45	0.76	Reference
No	1.0			1.0			1.0			

*Note*. Reference group is facilities not offering Driving Under the Influence (DUI) or other Criminal Justice (CJ) specialized programming. Odds ratios (OR)and 95% confidence intervals (CI) calculated from multinomial logistic regression with all listed variables entered simultaneously controlling for clustering effect of state location of facilities.

For location (Table 2), non-MSA location increased the likelihood of offering DUI specialized programming both with and without other criminal justice specialized programming. In contrast, MSA location increased the likelihood of offering other criminal justice specialized programming. Facilities in the Western region (13 states) compared to the other regions had an increased likelihood of offering other criminal justice specialized programming but only if within state clustering is ignored.

If the analysis had focused only on the provision of criminal justice specialized programming and ignored the separate category of DUI specialized programming, for-profit status would have increased the likelihood of offering any criminal justice specialized programming (OR = 1.46, 95% CI = 1.21, 2.76). When the two specialized programming are examined separately, for-profit status increased the likelihood that a facility would offer DUI specialized programming either with other criminal justice or without other criminal justice specialized programming. It did not increase the likelihood of offering other criminal justice specialized programming without DUI specialized programming (OR = 0.90, 95% CI = 0.79, 1.03).

## Discussion

The decision to offer, or advertise that a substance abuse treatment facility offers, specialized programming for DUI and other criminal justice offenders was common and associated with distinct facility characteristics. We believe this reflects that the market for substance abuse treatment for people with criminal justice involvement is large and shows no sign of declining. Fortunately, all the treatment facilities in the sample reported they "accept" criminal justice clients, indicating access to facilities. For maximal treatment success, however, specialized programming may be needed. It also means that policymakers – particularly those financing such services – should heed the quality and efficacy of these specialized programming.

The distinct characteristics associated with offering criminal justice specialized programming went beyond ownership and for-profit status to include funding sources, modality, population density and region. Furthermore, these associations were replicated over separate years, suggesting that the findings were not due to chance. Private ownership and for-profit status were independently associated with offering DUI specialized programming. Public funding was associated with offering other criminal justice specialized programming. These observed associations may reflect, as stated above, the positioning of the facility to increase visibility, or diversify clientele and possibly funding streams. As both target populations involve interface with criminal justice system and resources are scarce, it is important to examine the efficient use of resources. If so many for-profit enterprises find DUI programming attractive (including offering both types of specialize programming), are non-for-profit enterprises offering specialized programming for other criminal justice populations missing an opportunity to diversify clientele and funding sources? Likewise, are there regulations that restrict or encourage certain facilities to offer these specialized programming? Unfortunately, while using a national survey has the advantage of broadly describing associations, it does not provide answers on why it exists. It does reflect the lack of U.S. national restrictions or standards on facilities offering these specialized programming.

The association of DUI specialized programming with outpatient treatment and other criminal justice specialized programming (without DUI specialized programming) with non-hospital residential treatment is consistent with differing concerns regarding secure placement for public safety. With the overcrowding of prisons and jails, residential facilities may provide an alternative secure placement. However this raises other policy concerns and organizational issues: the role of treatment versus security; staff as professional clinicians or custodians; and evidence based practices versus criminal justice interventions.

The finding that the western region has a higher percentage of facilities offering other criminal justice specialized programming compared to the rest of the country when ignoring clustering is not as easily explained. The prison overcrowding is nationwide but there may be greater appreciation of the need for specialized programming or higher volume (e.g., methamphetamine admissions are higher in the west and they are more likely to be referred by the criminal justice system [21]). It also could reflect different regulatory environment or possibly targeted funding.

The positive association of offering either DUI or DUI and other criminal justice specialized programming with non-metropolitan locations could be due to limited treatment options in these areas. Treatment facilities may feel responsible or pressured by policymakers to offer services to all potential clients in the area. Alternately, it could reflect the lack of pressure to specialize (offering only a limited number of specialized programming). Finally, it is also possible that lower labor and land costs in non-metropolitan locations may facilitate the expansion of programming. Clearly this part of the analysis was exploratory and needs further attention. However, the association of urban locations with facilities offering specialized programming for other criminal justice offenders suggests efforts to align treatment location with areas of high demand.

This study is limited by lack of knowledge on the type or quality of programming constituting specialized programming for DUI and other criminal justice offenders as well as the number of clients enrolled in them. Additionally we could not examine ancillary services due to lack of knowledge of which clients were eligible to receive which services. Using a national survey can paint a broad perspective but more indepth studies would be needed to detail the regulatory and facility interface and organizational context. For example, facilities may admit a few criminal justice clients or may be devoted entirely to treating criminal justice clients. Lastly we can not explain the motivation for the observed associations. They may reflect reimbursement, but they could also reflect desire to please funders, regulations or requirements for treatment associated with each client population.

From a policy perspective, knowing the magnitude of community-based facilities currently providing treatment programming to DUI and/or other criminal justice clients makes it incumbent upon policymakers to join forces to examine them for efficacy, and explicitly incorporate these characteristics into strategies for workforce training and plans for treatment expansion. At the state and local level, knowledge of facilities' characteristics offering specialized programming informs training plans and assessment of treatment expansion. It also importantly allows review of state-specific regulations in the context of the national picture and lastly, predicts the impact of perturbations to current treatment system. At the facility level, the findings suggest different strategies for survival/expansion.

Movement towards treatment rather than incarceration for drug offenders [22,23], the current focus on reentry services [24], and the evidence that a continuum of care between the institution and community treatment enhances outcomes [25,26] may escalate the demand for other criminal justice specialized services. In California where Proposition 36 suddenly ballooned the number of individuals entering treatment through the courts, policymakers were faced with how to rapidly expand treatment capacity [22,23]. Although each county worked locally with known providers, familiarity with the type of facilities offering specialized programming might assist policymakers elsewhere when *planning *similar expansion or *predicting *the impact of large changes to the system.

## Conclusion

This study is a preliminary look at characteristics of facilities offering specialized programming to two groups involved in the criminal justice systems, those with a DUI and those with other offenses, with almost half of publicly and privately owned facilities in the U.S. offering either one or both of these specialized programming. The patterns observed seem to reflect real differences in, but do not address motivation for, decisions to report offering these specialized programming. These observed associations may reflect the positioning of the facility to increase visibility, or diversify clientele and possibly funding streams or regulations. As both target populations involve interface with criminal justice system, it is important to understand the motivation to offer specialized programming and from this knowledge, enhance survival of treatment facilities, disseminate efficacious programming and workforce training, and plan for sudden perturbations to the treatment system.

## Competing interests

The author(s) declare that they have no competing interests.

## Authors' contributions

Dr. Arfken contributed to the development, analysis and writing. Dr. Kubiak contributed to the development, analysis and writing.
